# Consensus Recommendations for Maintaining Neurorehabilitation Quality During Healthcare Crises: A Stakeholder-Informed Mixed Methods Study

**DOI:** 10.1177/00469580251390298

**Published:** 2025-11-27

**Authors:** Perrine Ferré, Diana Zidarov, Claude Vincent, Palak Vakil, Dahlia Kairy, Nayra Abdel Fattah, Gabriela Huerta Castro, Alison Lam, Joella Reev, Louis-David Beaulieu, Johanne Higgins, Marie-Hélène Milot, Marie-Hélène Boudrias

**Affiliations:** 1 School of Physical and Occupational Therapy, Faculty of Medicine and Health Sciences, M﻿cGill University, Montreal, QC, Canada; 2 Centre de recherche interdisciplinaire en réadaptation (CRIR) du Montréal métropolitain, QC, Canada; 3 Institut universitaire sur la réadaptation en déficience physique de Montréal (IURDPM) du CIUSSS-Centre-Sud-de-l’Île-de-Montréal, QC, Canada; 4 Université de Montréal, QC, Canada; 5 Université Laval, and Centre interdisciplinaire de recherche en réadaptation et intégration sociale (Cirris), Québec, QC, Canada; 6 Université du Québec à Chicoutimi, QC, Canada; 7 CIUSSS-Saguenay-Lac-St-Jean, Chicoutimi, QC, Canada; 8 École de réadaptation, Faculté de médecine et des sciences de la santé, Université de Sherbrooke, Sherbrooke, QC, Canada; 9 Centre de recherche sur le vieillissement, CIUSSS-de-l’Estrie CHUS, Sherbrooke, QC, Canada; 10 BRAIN Lab, Jewish Rehabilitation Hospital, CISSS-Laval, Laval, QC, Canada

**Keywords:** COVID-19, neurological rehabilitation, mixed-methods research, health services research, care quality

## Abstract

The COVID-19 pandemic significantly disrupted neurorehabilitation practices in Inpatient Rehabilitation Facilities (IRFs), providing an opportunity to examine crisis response strategies. This mixed-methods study examined the pandemic’s impact on quality of care through 3 complementary phases: (1) multivariable medical record analysis across 6 neurorehabilitation facilities in a pre-pandemic reference group (n = 134), a non-COVID group (n = 138), a COVID-positive group infected prior to admission ((COV+prior, n = 87) and a group infected during rehabilitation (COV+rehab, n = 36); (2) qualitative analysis of stakeholder consultations with patients (n = 26), staff members (n=55), and managers (n = 7); and (3) integration of quantitative and qualitative results to develop stakeholder and consensus-based recommendations (n = 12 participants). While non-COVID patients (n = 138) maintained pre-pandemic outcome levels, COVID-positive patients showed reduced functional independence at discharge, with distinct trajectories based on infection timing, even after controlling for status at admission. Patients infected during rehabilitation (n = 36) experienced longer stays and higher readmission rates compared to those infected pre-admission (n = 87). Qualitative analysis identified psychosocial, discharge planning, and resource management issues affecting both COVID-positive and non-COVID patients, emphasizing the impact of social isolation. Integration of findings led to 5 consensus-based recommendations: adaptation of COVID unit environments, family involvement, socialization promotion, safe care trajectory planning and flexible local management. These findings highlight the need for balanced approaches between infection control and rehabilitation quality during healthcare crises, guided by local leadership.

## Introduction

Over two-thirds of adults with neurological disorders—including stroke, brain tumors, and multiple sclerosis—require transfer to Inpatient Rehabilitation Facilities (IRF) following acute care.^1^ Neurological rehabilitation in IRF follows well-established quality standards of care that optimize physical, cognitive and emotional recovery through timely, intensive, and specialized care delivered by interdisciplinary teams in dedicated facilities.^2^ These standards emphasize a participatory approach involving patients and families, with specific protocols guiding standardized assessement^2^ and the rehabilitation trajectory.^3^ IRFs employ comprehensive quality indicators to monitor various aspects of care delivery,^4^ from admission timing to therapy intensity and functional outcomes, ensuring organizational efficiency and adherence to best practices in neurorehabilitation.^5^ Best practices include timely screening of depression and cognitive skills, as well as motor and cognitive assessments performed by rehabilitation professionals—including occupational therapists, physical therapists, speech pathologists, and psychologists/neuropsychologists.^2,3^ However, during a pandemic such as the COVID-19, the established practices can be significantly disrupted, forcing governments to introduce exceptional care measures. In Canada, on March 14th, 2020, the province of Quebec declared a Public Health Emergency^6^ becoming the most severely impacted province by COVID-19.^7^ The Ministère de la Santé et des Services Sociaux (MSSS) responded by implementing a comprehensive contingency plan,^8^ similar to approaches implemented internationally.^9^ This included designating specific COVID-19 IRFs in each region of the province to establish COVID units for all COVID-positive cases. These units accommodated 2 types of COVID-positive patients: those who tested positive for SARS-CoV-2 prior to rehabilitation admission and were transferred from acute care or non-designated rehabilitation centers, and those who acquired SARS-CoV-2 (COV+prior) nosocomial infection: while undergoing neurorehabilitation in the non-COVID units of the designated facility (COV+rehab). Healthcare teams in COVID units provided bedside treatments under strict infection prevention and control measures, including mandatory personal protective equipment and rigorous disinfection protocols. Meanwhile, non-infected patients (non-COVID) received care in regular units using modified protocols, including preventive isolation during admission or outbreak periods, and restricted visitor access. The challenges were compounded by COVID-19′s multisystemic manifestations, particularly fatigue and respiratory complications, which could significantly impact both general health status^10^ and neurological condition severity.^11^

This unprecedented situation created a unique opportunity to evaluate health system performance and quality improvement capacity during crises. Our research group’s preliminary findings^12^ revealed concerning trends, including extended lengths of stay for stroke patients in COVID units and greater functional dependency at discharge, indicating significant impacts on rehabilitation effectiveness. The timing of COVID-19 infection proved crucial, with better outcomes for patients who acquired it before admission versus during rehabilitation. Studies of nosocomial outbreaks in neurorehabilitation settings found that COVID-19 infection during rehabilitation reduced functional independence by up to scores 8.9 points on the Functional independence measure (FIM).^13^ Additionally, simply being in an outbreak ward negatively affect functional outcomes, regardless of infection status, suggesting that environmental and operational disruptions during outbreaks may independently impact rehabilitation effectiveness.^14^ While valuable, these studies have relied predominantly on quantitative measures, which fail to fully capture the complex interplay of factors affecting rehabilitation care delivery during crises. This methodological limitation has constrained our ability to develop truly comprehensive strategies for maintaining rehabilitation quality during future systemic disruptions. International qualitative research has enriched our understanding by incorporating diverse stakeholder perspectives, revealing how the pandemic has transformed rehabilitation program structures through caregivers’^15^ and practitioners’ experience^16^ and identified effective coping strategies through the roles of professionals.^17,18^

However, few studies have integrated both quantitative measures and qualitative stakeholder insights to comprehensively inform future crisis response strategies. This integration is crucial for developing a more nuanced understanding of rehabilitation needs during healthcare disruptions. This study uniquely contributes to healthcare crisis management by integrating quantitative rehabilitation outcomes with qualitative stakeholder perspectives across different COVID-19 exposure scenarios, resulting in the first stakeholder and evidence-informed guidelines specifically designed to maintain neurorehabilitation quality during healthcare disruptions. The main objectives are threefold: (1) To compare quality indicators of post-acute inpatient neurorehabilitation across 4 patient groups: COVID unit patients (with prior or rehab-acquired infection), non-COVID unit patients, and a pre-pandemic reference group. (2) To analyze stakeholders’ perceptions on how the pandemic affected rehabilitation quality in both COVID and non-COVID units and their recommendations for quality improvement. (3) To integrate these findings into consensus-based, actionable recommendations for maintaining rehabilitation quality during future healthcare crises. We hypothesized that COVID-positive patients (both COV+prior and COV+rehab) would show reduced quality indicators during their neurorehabilitation stay in designated IRFs, with COV+rehab patients experiencing the most significant decline in outcomes. We also expected that stakeholder interviews would provide deeper insights into how and why pandemic-related changes affected rehabilitation quality—insights that medical charts alone could not capture. These combined findings would support the creation of practical recommendations for maintaining care quality during crises.

### Methods

#### Conceptual Framework

**Figure 1.**
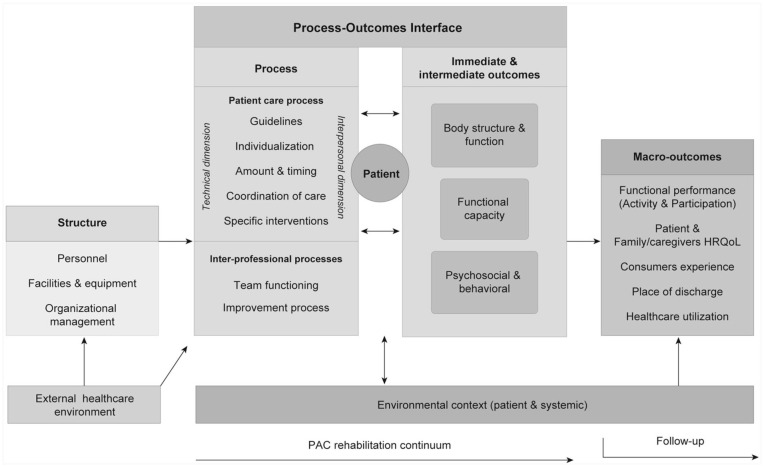
PAC-rehabilitation quality framework. *Source.* Jesus & Hoenig, 2015.^19^ *Note.* Copyright of the © 2015 American Congress of Rehabilitation Medicine, originally published by Elsevier Inc., and reused with permission (license number 5816060151728).

This study used the post-acute care (PAC) rehabilitation quality framework,^19^ a patient-focused framework that is adapted for post-acute rehabilitation (Figure 1). Inspired by Donabedian^20^ and the International Classification of Functioning, Disability and Health,^21^ the framework relates constituents of patient-care outcomes to its processes and structures.

#### Study Design and Setting

**Figure 2.**
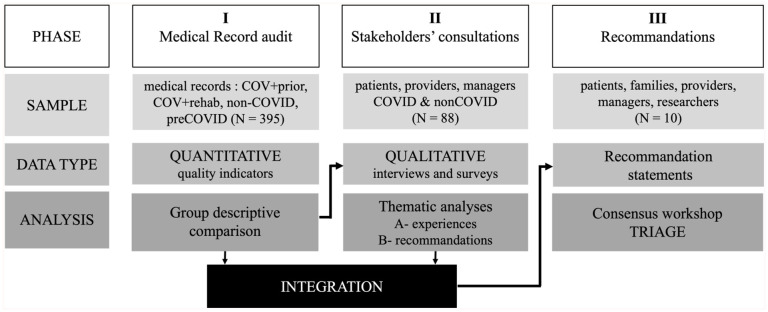
Overview of study design and process.

We implemented a 3-phase sequential explanatory mixed-methods study to investigate the impact of the COVID-19 pandemic on rehabilitation quality and develop consensus-based recommendations for future healthcare crises. This study adhered to the GRAMMS guidelines^22^ for mixed methods research reporting. Figure 2 synthesizes the study design and process.

The study setting consists in 6 rehabilitation facilities designated to handle COVID-19 patients, selected from 14 eligible sites participated in the study. Eight sites were excluded as they either did not admit COVID-19 positive neurological patients during the study period or were classified as non-traditional rehabilitation institutions. The Center for Interdisciplinary Research in Rehabilitation of Greater Montreal (CRIR) granted ethical approval (Supplemental File) for both study phases (Phase I: MP-50-2022-1297; Phase II: MP-50-2022-1544). In conformity with ethical assessment, authorization to access the medical charts was granted by each participating facility in accordance with local legislation. For the second and third consultative phases, verbal consent was obtained from patients contacted by phone and documented in the interviews verbatim. Staff members and managers invited via email by their organization provided written consent.

### Data Collection

#### Phase I—Comparison of Medical Records

We conducted a comprehensive medical records review that included all patients admitted to COVID units between March 2020 and July 2021. This finite sample comprised patients transferred from another site who tested positive before admission (COV+prior) and those who tested positive during their stay in rehabilitation facilities (COV+rehab). COVID-19 diagnosis was confirmed through RT-PCR testing. Our statistical analysis compared clinical indicators from these medical records with those from a matched group of non-COVID patients admitted during the same period (non-COVID) and a pre-pandemic reference group of patients admitted in the year before the pandemic (pre-COVID, March 2019-February 2020). To minimize selection bias, admission timing was distributed across the study period with balanced sex ratios between groups. Trained research assistants extracted data on performance and quality indicators.

We selected 12 indicators traditionally collected by rehabilitation organizations as part of routine performance and quality assurance,^4^ falling into 4 categories of the PAC rehabilitation quality framework:

- Sociodemographic and medical characteristics at admission: age, sex, comorbidities, time post onset (time from admission to acute care until admission to rehabilitation), the Functional Independence Measure (FIM) score at admission, a typical and validated index of functional autonomy^23^
- Rehabilitation structure and process indicators: treatment dose (the number of sessions attended by the patient with any staff member divided by length of stay), interdisciplinary involvement (the number of rehabilitation disciplines involved in the patients’ care, such as occupational therapy, physical therapy, respiratory therapist), length of stay (days between admission and discharge as documented in medical records)
- Functional outcome at discharge: the Functional Independence Measure (FIM) score at discharge)
- Macro-outcomes: rehospitalization events, occurrence of pressure injuries and delirium episodes documented in the medical records.

### Statistical Analysis

#### Descriptive Analysis

To provide an initial descriptive comparison across all indicators, unadjusted group-level analyses were conducted using the Kruskal-Wallis test for continuous or ordinal variables and the Chi-square test for categorical variables. The false discovery rate (FDR) was controlled using the Benjamini-Hochberg procedure, with a significance threshold of *q* = 0.05. Post hoc pairwise comparisons versus the PRE_COVID group were performed using Dunn’s test (for Kruskal-Wallis results) or pairwise Chi-square tests, with FDR correction applied. This approach was chosen to balance the control of false positives (Type I errors) with the preservation of statistical power while managing multiple comparisons.^24^ Missing data were handled using pairwise deletion (available-case analysis) to maximize the available sample size for each analysis. Missing data rates ranged from 0% to 33% across variables (detailed counts provided in Supplemental File 1). These exploratory comparisons are reported to complement adjusted multivariable models and provide a complete view of potential group differences.

#### Multivariate Analysis

Multivariable models were fitted for process and outcome quality indicators with low missingness (<10%): treatment dose, interdisciplinary team involvement, number of assessments, length of stay, in-hospital relapse, pressure sores, and episodes of delirium. Each indicator was modeled independently using regression models appropriate to its distribution (logistic regression for binary outcomes, quantile regression for skewed continuous variables). All models were adjusted for the individual’s characteristics at admission, represented by 5 pre-specified confounders: age, sex, time post-onset, number of comorbidities, and baseline functional status (FIM at admission). Covariates were selected based on having <15% missing data to ensure reliable imputation. Missing data were addressed using multiple imputation by chained equations (MICE) with 10 imputed datasets under the missing-at-random assumption, with results pooled using Rubin’s rules. Given the moderate sample size, particularly for the COVID admission group (n = 36), models were kept parsimonious without interaction terms to preserve statistical power and avoid overfitting. Missing data percentages are reported in Supplemental Table 1. All statistical analyses were performed using R (version 2024.09.1+394), with packages including “stats,” “dunn.test,” “mice,” and “multcomp.”

##### Phase II - Stakeholder Perspectives From Interviews and Surveys

To understand how rehabilitation quality was experienced during the pandemic, we developed and validated custom interview guides and surveys based on Phase I findings and the PAC rehabilitation quality framework, as there were no existing questionnaires validated for rehabilitation and COVID-19. The surveys underwent pre-testing with 3 patients and 5 rehabilitation professionals to ensure the questions were understandable, relevant, and comprehensive, then professionally reviewed for bilingual accuracy (French and English are the 2 official languages in Canada). Questions covered areas uncovered by phase I chart auditing and was supplemented by areas of the PAC rehabilitation quality framework that are not documented in medical charts (eg, personnel, facility, equipment, discharge coordination, team functioning, and guidelines). Open-ended questions encouraged expanded responses throughout the interview-survey, especially for questions specifically pertaining to recommendations (See Supplemental Appendix for complete interview guides).

#### Data Collection

From June 2022 to January 2023, we collected data through:

- Structured phone interviews with neurological patients (n = 24). Neurological patients were recruited by phone through convenience sampling from Phase I. Participants needed to be able to respond to questions in French or English or have a family member available to support them.
- Semi-structured virtual interviews with program managers (n = 12). These interviews lasted 45 to 60 min and were audio-recorded with consent.

Online questionnaires for staff members (n = 36) to accommodate their clinical duties.

#### Qualitative Analysis Framework

We used a multi-layered coding framework that included structural coding based on our interview guide, to organize data by stakeholder group (patient, professional, manager) and question/topic and descriptive coding to capture content areas (eg, infection control measures, telehealth use, staffing issues).

Using NVivo and Excel for thematic analysis, we coded transcripts until reaching theme saturation, which we defined as 3 consecutive interviews yielding no new codes. Two pairs of researchers independently conducted inductive theme generation and validation, with regular consensus meetings to resolve discrepancies. The thematic analysis followed the recommendations and phases described by Thomas and Harden^25^: (1) verbatim coding, (2) development of descriptive topics, and (3) generation of analytical themes and pattern extraction.

The first author reviewed all themes related to the stakeholder’s experience and performed deductive inferencing, associating themes with relevant categories of the PAC rehabilitation quality framework (part A). The answers pertaining to recommendations were subjected to a separate theme analysis (part B). Transformation was performed using frequency analysis as defined by the number of participants who mentioned each code. To enhance transparency, we included representative quotations for each major theme. Finally, we integrated quantitative and qualitative findings through triangulation using a joint display approach. This method highlighted convergences, divergences, and expansions between data sources,^26^ stakeholder groups, and units of care (COVID vs non-COVID).

##### Phase III—Consensus-Based Recommendations

The final study phase integrated findings from previous phases through a consensus-building workshop, planned in mid-2023 and conducted in January 2024. This workshop aimed to establish priority recommendations to guide rehabilitation care system decision-makers during future healthcare crises. We employed the TRIAGE^27^ method (Technique for Research of Information by Animation of a Group of Experts), a constructivist approach that acknowledges diverse contextual realities shaped by participants’ experiences. To facilitate meaningful dialog, we limited participation to 10 individuals. The research team was represented by the first and last authors who served as content experts, contextualizing findings and guiding the consensus process. We invited all 7 unit managers, who were asked to nominate healthcare professionals from their teams and arrange clinical release time. Additionally, we selected 7 patients who had participated in Phase II interviews using a semi-random approach that ensured representation from both COVID and non-COVID units across different facilities. Recruitment concluded once we reached our predetermined participant limit.

Participants received key statements and prioritization criteria 1 week before the workshop. The assessment criteria evaluated whether each proposed solution was: (1) necessary for rehabilitation effectiveness (supporting autonomy progress), (2) actionable within management constraints (budget, length of stay, staffing) and safety requirements, (3) beneficial to patient and family well-being, and (4) essential for healthcare professionals’ working conditions.

Following the TRIAGE methodology guidelines as described by Gervais & Pépin (2002), the 1-round workshop employed the prescribed 5-component visual system to organize proposals: “Collective Memory” (initial considerations), “Selection” (chosen recommendations), “Trash” (eliminated proposals), “Refrigerator” (items requiring further discussion), and “Veto” (formal objections). A professional knowledge broker facilitated the virtual workshop using visual tools to guide the process, beginning with a “Collective Memory” exercise where participants validated their understanding of the key statements. This was followed by collaborative discussion to sort, reformulate, and prioritize recommendations. We considered content saturation reached when discussions no longer generated new themes or ideas. After the workshop, the knowledge broker compiled a synthesis of unanimously agreed statements—with no veto—along with raw data. Team members (PF, JH, DK, MHB) then conducted a final review to ensure the recommendations were clear and actionable.

## Results

Results are presented for the 3 phases of the mixed-methods design (I, II, III).

### Comparison of Medical Records Indicators Between Groups of Patients

#### Descriptive Analysis

A total of 396 medical records from participating institutions were audited. One participant without COVID-19 was excluded due to a length of stay exceeding 200 days. Among the 122 patients with COVID-19, the majority (n = 87) contracted the virus during their stay in the designated IRFs (nosocomial infection), while 36 contracted it before admission, such as during or before acute care or in a non-designated IRF they were transferred from.

As presented in Table 1, the characteristics of the patients at admission showed no group differences in regards to age (*H*(3) = 5.86, *P* = .442) or sex (χ²(3) = 2.691, *P* = .442), and baseline number of comorbidities χ²(3) = 1.50, *P* = .680). The general function, as assessed by FIM at admission, was significantly lower for the COV+rehab group than the pre-COVID reference group (*H*(3) = 16.36, *P* < .001).

**Table 1.** Socio-Demographics and Medical Characteristics of Patients at Admission.

Variables	COV+prior	COV+rehab	Non-COVID	Pre-COVID	Uncorrected *P* values (main effect)	FDR corrected *P* values (main effect)
N	36	87	138	134		
Mean age (std)	74 (13)	75 (12)	72 (13)	71 (13)	.118	.16
Sex f/m (%)	16/20 (44%/56%)	45/42 (52/48%)	66/72 (48/52%)	55/79 (46/54%)	.442	.53
N comorbidities (std)	2.75 (1.14)	2.81 (1.09)	2.82 (1.21)	2.95 (1.28)	.682	.68
FIM admission (std)	68.21 (25.95)	**65.09 (20.32)**	75.78 (21.61)	77.29 (24.43)	.001	**.002**

*Note.* Values are presented as mean ± standard deviation. The *P*-values shown represent the overall group effect from Kruskal-Wallis or Chi-square tests, comparing all groups to the pre-COVID reference group. Values in bold indicate significant differences (*q* < 0.05) between that specific group and the reference group in post hoc analyses using Dunn’s test or Chi-square tests with Benjamini-Hochberg correction for multiple comparisons.

In regard with rehabilitation process indicators, the Kruskal-Wallis test revealed significant differences in the number of various therapists involved in the patients’ care (*H*(3) = 56.27, *P* < .001). The COV+prior group had significantly fewer (*M* = 3, *Z* = −5.39, *P* < .001) and the COV+rehab nosocomial group had more therapists involved (*M* = 6, *Z* = 3.19, *P* < .001) compared to the PRE-COVID group (*M* = 5, *Z* = 1.71).

The reporting of functional performance as assessed by standardized assessment tools was significantly lower (*H*(3) = 41.07, *P* < .001) across all groups during the pandemic, with COV+prior (*M* = 9, *Z* = −5.93, *P* < .001), COV+rehab (*M* = 13, *Z* = −2.94, *P* = .002) and nonCOVID groups (*M* = 13, *Z* = −4.21, *P* < .001) showing lower use of standardized tools compared with the pre-COVID group (*M* = 15, *Z* = 5.24).

Length of stay (*H*(3) = 69.91, *P* < .001) and time post onset (*H*(3) = 37.29, *P* < .001) significantly differed between groups. With an average of 79 days, the COV+rehab group had longer (*Z* = 4.88, *P* < .001) and COV+prior (*M* = 30 days) had shorter length of stay (*Z* = −4.24, *P* < .001) than the pre-COVID group (*M* = 53, *Z* = 30.88). Both COV+rehab group (*M* = 28 days, *Z* = 3.42, *P* < .001) and COV+prior had longest time post onset (*M* = 53 days, *Z* = 5.67, *P* < .001) than the pre-COVID group (*M* = 23, *Z* = 23.31). Treatment dose was significantly lower in COV+prior (*M* = 1, *Z* = −4.74, *P* < .001) compared to pre-COVID (*M* = 2, *Z* = 0.6).

Significant differences were found regarding hospital readmission during rehabilitation (χ² (3) = 28.19, *P* < .001) with 42% of COV+rehab patients requiring acute care readmission against 20% pre-pandemic (χ² (3) = 11.28, *P* = .002). Significant main group differences were found regarding incidence of delirium (χ² (3) = 12.41, *P* = .011), but no differences were found between COVID and pre-COVID groups (*P* > .05). Pressure sores incidence showed no significant differences between groups (χ² (3) = 1.68, *P* = .68).

Finally, groups significantly differed in their FIM score at discharge (*H*(3) = 11.71, *P* < .015) with both COV+prior (*M* = 81, *Z* = −2.41, *P* *=* .024) and COV+rehab (*M* = 95, *Z* = −2.86, *P* *=* .041) groups having lower scores than the pre-pandemic group (*M* = 101, *Z* = 21.43).

All process and outcome measures are reported in Table 2.

**Table 2.** Descriptive Analysis: Group Comparison of Medical Charts’ Indicators.

PAC quality category	PAC quality category	Chart indicator	COV + PRIOR	COV + REHAB	NON-COVID	PRE-COVID	FDR corrected *P* values (main effect)
		N	36	87	138	134	
Structure	Personnel	Rehabilitation disciplines	**3.11 (1.41)**	**5.67 (1.59)**	4.64 (1.71)	4.93 (1.70)	<**.001**
Process	Guidelines	Standardized assessments	**9.19 (4.97)**	**13.02 (5.48)**	**12.62 (4.96)**	15.37 (5.24)	<**.001**
	Amount and timing	Intervention dose	**1.06 (0.35)**	1.49 (0.55)	1.58 (0.61)	1.51 (0.60)	<**.001**
		Length of stay	**30.03 (18.96)**	**79.43 (36.78)**	49.34 (37.28)	53.49 (30.88)	<**.001**
		Time post onset	**53.23 (40.04)**	**27.83 (17.23)**	24.26 (20.31)	22.82 (23.31)	<**.001**
Outcomes	Body structure and function	FIM score discharge	**80.96 (39.84)**	**95.03 (22.46)**	102.53 (21.82)	100.75 (21.43)	**.015**
Macro-outcomes	Healthcare utilization	Hospital readmissions (relapse)	3 (8%)	**35 (42%)**	19 (14%)	24 (20%)	<**.001**
		Deliriums	1 (3%)	18 (21%)	10 (7%)	17 (13%)	**.011**
		Pressure sores	8 (22%)	15 (17%)	19 (14%)	23 (17%)	.68

*Note.* Values are presented as mean ± standard deviation. The *p*-values shown represent the overall group effect from Kruskal-Wallis or Chi-square tests, comparing all groups to the pre-COVID reference group. Values in bold indicate significant differences (*q* < 0.05) between that specific group and the reference group in post hoc analyses using Dunn’s test or Chi-square tests with Benjamini-Hochberg correction for multiple comparisons. *P*-values have been adjusted for multiple comparisons using the False Discovery Rate (FDR) method.

#### Multivariate Analysis

Multivariable analysis revealed significant group differences across process indicators. COVID admission patients demonstrated the shortest rehabilitation stays (reference group), while all other groups showed significantly longer stays (adjusted β = +51.0 days, all *P* < .001). COVID nosocomial patients exhibited the highest risk profiles or rehospitalization, with eightfold increased odds of relapse during their rehabilitation stay (OR = 8.39, 95% CI: 2.20-32.09) and 13-fold increased odds of delirium (OR = 13.13, 95% CI: 1.51-114.10) compared to COVID admission patients (Table 3).

**Table 3.** Adjusted Multivariate Model Results.

Chart indicator	Group compared to PRE_COVID	Estimate (OR/beta)	95% CI	*P* (FDR adjusted)
Dose_Tx	COVID_adm	−0.42	−0.65, −0.19	**.001**
Dose_Tx	non-COVID	−0.42	−0.07, 0.21	.415
total_N_tests	COVID_noso	−5.14	−3.72, −0.88	.**005**
relapse	COVID_noso	0.28	1.22, 4.43	.**028**
delirium	COVID_adm	0.11	0.01, 0.96	.097

*Note.* This table presents the adjusted effects of patient admitted during the COVID pandemic (COVID_adm, COVID_noso, non-COVID) compared to the reference group (PRE_COVID) on various quality indicators. Values in bold indicate significant differences (*q* < 0.05) between that specific group and the reference group. Estimates are reported as β coefficients for linear models and odds ratios (ORs) for logistic models, with corresponding 95% confidence intervals (CIs). *P*-values have been adjusted for multiple comparisons using the false discovery rate (FDR) method.

### Stakeholders’ Consultations

Out of the original list of 261 **patients** admitted during the pandemic period, 26 (proportion: 10%; 95% CI: 0, 0.21) completed the phone interviews while others either refused to participate (N = 47), were unreachable at the provided number or deceased (N = 205), cognitively impaired or not able to respond in French or English (N = 9). The interviews with the participating patients lasted between 10 and 60 min. Of all participants living independently, 58% resided with other people in their own or family homes while 42% lived alone. In total, 72% were retired, 20% worked and 4% were unemployed. **Staff members** participants were invited to participate if they were a rehabilitation professional at 1 of the participating designated COVID-19 rehabilitation centers, have provided rehabilitation during the period between March 2020 and July 2021. Online questionnaires were completed by 55 out of approximately 144 staff members (among physiotherapists, occupational therapists, speech-language pathologists, neuropsychologists, psychologists) to whom an email invitation was sent by the coordinator/program chief of the participating facilities (proportion: 39%; 95% CI: 0.28,0.48). It took on average, 29 min to complete the questionnaire. The participating respondents included physiotherapists (61%), occupational therapists (37%) and speech-language pathologists (2%). Most were specialized in neurology (49%). Others reported a speciality in physical health (14%) or traumatology (16%) but were assigned to the COVID unit during the first waves of the pandemic. The median experience in rehabilitation was 15 years (range: 1-25 years). Out of 9 **managers** invited, 7 accepted to participate (78%; 95% CI: 0.78, 0.97). Four of them oversaw a non-COVID neurorehabilitation unit and 3 others a COVID unit during the first waves of the pandemic. Interviews lasted between 45 and 75 min.

#### Stakeholders Experience

Many commonalities emerged across stakeholder groups, aligning with the 3 main components of the PAC-rehabilitation quality framework. While experiences were mostly consistent across unit types—with most sub-themes encompassing both COVID and non-COVID units—some sub-themes were particularly prominent in COVID units, as highlighted in Table 4.

**Table 4.** Stakeholders Perspectives: Comparative Analysis Themes and Sub-Themes in COVID and Non-COVID Units.

Theme	Subtheme	Stakeholder groups and illustrative quote	COVID unit (n)	Non-COVID unit (n)	Total frequency
1. Resource and infrastructure challenges (structure)	Material and human resources limitations	Staff members “*When we had to treat in patient rooms during buffer zone, the space was not adequate for rehabilitation. On the other hand, when we had a closed hot unit, we were able to bring patients to the department*”	22	10	32
		Managers “*we don’t have any wiring in the walls. We have no oxygen. We do not have a suction device. And they asked us to keep our COVID patients. It was a disaster.*”	3	4	7
	New challenges in organizational management	Managers “*It was horrific because I had to send kids who are 22-23* years *old. [. . .] There was nobody there to train her until she found someone, until she figured out what she was doing*”	3	3	6
	Professional autonomy undermined	Staff members “*I had to treat patients from four programs at the same time. I did not have the expertise*”	6	0	6
2. Care delivery challenges (process)	Insufficient care	Patients “*I didn’t have enough physiotherapy or occupational therapy for my liking because of the confinement*”	6	6	12
	Gratefulness and understanding	Patients “*I don’t blame them. [. . .] It’s normal to be afraid to enter the room if they are not properly equipped.*”	4	4	8
	Standards of care limitations	Staff members “*Anything hobby-related was impossible due to the lack of equipment, especially in the beginning. As an OT this is essential.*”	12	8	20
		Managers “*Everything* that is *technological is in the evidence [. . .]. It is certain that there have been impacts [on quality]*”	3	3	6
	Difficult discharge planning	Patients “*The center did not transfer my file [. . .] and I have little access to any resources. My caregiver had to do it all on her own.*”	5	7	12
		Managers “*There were waiting lists, there was no place. I know that our patients were going to concentration camps, waiting, places which were waiting for long term care homes.*”	3	4	7
		Staff members “*At times, it was impossible to assess functional tasks outside the room. Home visits were also limited, making discharge planning more complex.*”	4	7	11
	Teamwork adaptations	Staff members “*The challenges were numerous but with teamwork and the support of our immediate superiors the impact of several limiting elements was able to be reduced.*”	5	3	8
		Managers “*And that willingness to work as a team, to do different things and to learn as you go - even with all the uncertainty - it helped.*”	3	4	7
3. Psychosocial impact and functional outcomes	Psychosocial needs	Patients “*We should also allow the family inside [. . .] we need human contact, more than standing at the door, especially after having survived a stroke*”	10	7	17
		Staff members “*The isolation increased the agitation, disorganization of some clients who had cognitive difficulties and could not understand or follow the precautions of staying for 14* days *in their room.*”	11	5	16
		Managers “*Cognitively speaking, in terms of anxiety, in terms of signs of depression and signs of isolation, it was much more fragile, [. . .] it’s major, we also added general deconditioning to the after-effects of stroke.*”	3	3	6
	Isolation effects	Patients “*A lot of isolation and complexity with the fear of COVID and their protocols. I felt like I was in prison.*”	8	0	8
		Managers “*Discharge was given without a safety net*”	3	4	7

*Note.* This table presents a thematic analysis of stakeholder experiences in rehabilitation units during the COVID-19 pandemic. Data are presented as raw frequency counts (n) of reported experiences by different stakeholder groups (rehabilitation practitioners, managers, staff, and patients), accompanied by representative quotes. The analysis is organized around 3 main themes corresponding to categories of the PAC-rehabilitation quality framework. In red: citation from a COVID unit participant, in blue: citation from a non-COVID unit participant.

##### Resource and Infrastructure Challenges (Structure)

Material and Human Resources limitations: While both units reported resource challenges, rehabilitation practitioners in COVID units reported more frequently (n = 22 vs n = 10) struggling with staff shortages or inadequate infection protection equipment (eg, N95 masks not available). Managers across both units (n = 3 vs n = 3) highlighted infrastructure limitations, particularly in older buildings lacking technological capabilities.

New challenges in organizational management: Management perspectives revealed significant operational challenges regardless of unit designation (n = 3 vs n = 3). Key issues included implementing evolving directives and rapid restructuring requirements. That created workforce instability and forced rapid and sometimes difficult decisions, such as reassigning a professional to a new unit or facility based on load-balancing, without the proper expertise or support. A particular challenge emerged in adapting acute or long term care focused guidelines that were received to rehabilitation settings, especially regarding family visit restrictions, considering the emotional and cognitive vulnerability of the neuro-stroke population.

Professional autonomy undermined: Unique to COVID units (n = 6 vs n = 0), staff reported compromised professional autonomy when required to treat patients outside their specialty areas as the COVID unit admitted all diagnoses. Some were also instructed to minimize room entries and duration of therapy sessions to reduce virus transmission risk. Some disciplines (eg, social workers, psychologists, speech pathologists) were temporarily restricted from providing care in the COVID unit and had to wait for patient transfer to a non-COVID unit, while others were directed to use telerehabilitation exclusively, with iPads loaned to patients by professionals who were permitted to enter the zone (eg, nurses, physiotherapists).

##### Care Delivery Challenges (Process)

Care Quality: Patients across both units reported similar rates of insufficient care (n = 6 vs n = 6), (eg, wanting more frequent therapy sessions, feeling neglected for long periods) while acknowledging staff dedication (n = 4 vs n = 4) given the challenging circumstances. Limitations in usual standards of care and evidence-based practices were more pronounced in COVID units (n = 12 vs n = 8) according to rehabilitation practitioners. In particular, the limitation in functional activity practice (e.g.: walking, cooking) and equipment use impacted both standardized assessments and interventions. Some rehabilitation practitioners from the COVID units additionally reported that full-body equipment limited physical and social interactions.

Discharge Planning: Both units experienced similar challenges with discharge preparation experienced by patients (COVID: n = 5 vs non-COVID: n = 7) who felt unprepared, with family members having to handle communication, education (eg, medication, safety, setup), and everyday support, with minimal community support. Care coordination issues were reported by both staff members (n = 4 vs n = 7) and managers (n = 3 vs n = 4), who cited constantly changing directives, inconsistencies in admission rules between institutions, and limited community resources. Managers and staff members both noted that some patients were discharged earlier than usual—either to free up beds or because patients and families were eager to leave the hospital.

Teamwork Adaptations: While staff (n = 5 vs n = 3) and managers (n = 3 vs n = 4) of both units reported negative changes in team functioning because of the frequent bed transfers, staff reallocation and communication challenges, both units also demonstrated positive adaptations as they expressed a shared sense of purpose and professional accomplishment. The COVID unit environment especially fostered team cohesion with more flexible professional roles and creative problem-solving. Since all patients were COVID-positive with no risk of cross-contamination, the unit’s unique structure enabled staff to implement local initiatives for patient socialization and physical activity—such as setting up a gym in an unused room or organizing Bingo games in the hallway.

##### Psychosocial Impact and Functional Outcomes

Psychosocial Needs: Patients from both units reported unmet psychosocial needs (COVID: n = 10 versus non-COVID: n = 7), in particular feelings of loneliness and boredom with room isolation and little contact with their families. Management across both units (n = 3 vs n = 3) reported concerns regarding psychosocial distress among patients, with consequences of room isolation including lack of motivation to participate in therapies, depression, and cognitive disorientation—sometimes escalating to delirium. COVID units showed higher frequencies of staff-reported concerns (n = 11 vs n = 5), as staff dedicated to the COVID units felt ill-equipped to provide adequate patient support.

Isolation Effects: COVID unit patients uniquely reported isolation’s impact on their functional progress (n = 8 vs n = 0), citing limited professional interactions and COVID-related symptoms such as fatigue as key barriers to rehabilitation participation. Managers were the only ones who reported impacts of these changes on patients’ functional outcomes during this period (n = 3 vs n = 4), and they partly related this to the fact that evidence-based practice was not always feasible. Rehabilitation practitioners reported similar issues (n = 12 vs n = 8). Managers acknowledged that the circumstances prevented full assessment and practice of functional activities as before, resulting in patients being discharged with lower functional gains.

#### Integration of Quantitative and Qualitative Findings

Integration of quantitative and qualitative data using a joint display based on the PAC rehabilitation framework revealed several key findings across structure, process, and outcome domains (Table 5).

**Table 5.** Joint Display Integration of Quantitative Metrics (QUAN) and Stakeholder Experiences in COVID and Non-COVID Rehabilitation Units (QUAL) by Quality Category of the PAC Rehabilitation Framework.

Quality category	COVID units	Non-COVID units
Structure	Significant QUAN differences	
	COV+prior: fewer disciplines (*M* = 3) COV+rehab: more disciplines (*M* = 6)	Number of disciplines similar to pre-COVID (*M* = 5)
	QUAL findings	
	More material and human resources limitations according to staff members Professional autonomy undermined according to staff members Managers report challenges in organizational management	Resource constraints reported by staff members, to a lesser extent No report of professional autonomy issues Managers report challenges in organizational management
Process	Significant QUAN differences	
	Lower standardized assessment reporting (M = 9–13) Longer length of stay for COV+rehab (*M* = 79 days), shorter length of stay for COV+prior (*M* = 30 days) Lower treatment dose lower for COV+prior	Lower standardized assessment reporting (*M* = 13) Baseline length of stay (53 days)
	QUAL findings	
	Patients report insufficient care More frequent changes reported in care standards by staff members Similar discharge planning difficulties reported by patients, staff and managers	Fewer changes in care standards according to staff members Similar discharge planning difficulties reported by patients, staff and managers
Outcomes	Significant QUAN differences	
	Lower FIM scores at discharge (COV+prior: *M* = 81, RA: *M* = 95)	FIM scores similar to pre-pandemic levels (*M* = 101)
	QUAL findings	
	Psychosocial needs reported by rehabilitation professionals, managers and patients Isolation effects reported by patients and managers	Psychosocial needs reported—to a lesser extent—by rehabilitation professionals, managers and patients No isolation effects reported by patients
Macro-outcomes	Significant QUAN differences	
	Higher hospital readmission (42% for COV+rehab) and deliriums (21% for COV+rehab)	Lower hospital readmission (20%) and deliriums (7% for COV+rehab)

Structure findings showed significant variations in care delivery. COV+rehab patients received care from a higher number of disciplines throughout their stay, while COV+prior patients experienced reduced disciplinary involvement (*M* = 3 vs pre-COVID baseline *M* = 5). Stakeholders identified structural recommendations including enhanced treatment materials in confined spaces, improved communication equipment, and adapted physical environments. Resource limitations were reported more frequently in COVID units compared to non-COVID units by staff members (22 vs 10), as well as professional autonomy issues (6 vs 0) highlighting the need for improved employee working conditions under pandemic constraints and especially for the staff dedicated to COVID units.

##### Stakeholder’s Recommendations

Since no significant differences emerged between COVID and non-COVID units, recommendations were consolidated under 12 sub-themes (Table 6).

**Table 6.** Sub-Themes Related to Recommendations to Support Quality Services in Times of Crisis.

Recommendation sub theme	Total frequency	Staff (N = 55)	Managers (N = 7)	Patients (N = 26)	Illustrative quote
Equip confined spaces with treatment materials	42 (48%)	39 (71%)	3 (43%)	0 (0%)	“*And I think the investment in technology must be done before we necessarily need it*” *(manager)*
Adapt infection prevention rules to local facility context	34 (39%)	24 (44%)	7 (100%)	3 (12%)	“*We were always wondering which rules to follow - the rules of acute care facilities or those of long term care. Should we have something specific for rehab facilities?*” *(manager)*
Ensure continuity of care post-rehabilitation	25 (28%)	2 (4%)	0 (0%)	23 (89%)	“*The suspension of outings, weekend leaves home. It’s something that can go unnoticed in other sectors of activity where the length of stay is shorter and it’s less essential, but in our case [in rehabilitation], for some users, it created issues during the final discharge*” *(staff member)*
Alleviate boredom and isolation	23 (26%)	5 (9%)	4 (57%)	14 (54%)	“*We need human contact, more than just staying in front of the door, especially after surviving a stroke*” *(patient)*
Improve employee’s satisfaction and working conditions	21 (24%)	18 (33%)	3 (43%)	0 (0%)	“*I had to treat patients from four programs at the same time. So I didn’t have the expertise to treat these patients optimally. But I had them do mobilization activities*” *(staff member)*
Offer more care and exercises during rehabilitation	16 (18%)	0 (0%)	1 (14%)	15 (58%)	“*With staff on leave, I went 3 days without exercise or supervision. I would have liked more services. I would have had more success*” *(patient)*
Adapt the physical environment to create a living space	13 (15%)	9 (16%)	2 (29%)	2 (8%)	“*The isolation conditions are not rehabilitation conditions, as we cannot reproduce functional activities having access only to the bed*” *(staff member)*
General satisfaction with therapeutic relationships	10 (11%)	0 (0%)	0 (0%)	10 (39%)	“*The kindness of the staff is extraordinary. The people at the center are dedicated and you feel like you’re a person and not a number. I recommend continuing like this!*” *(patient)*
Challenge the trajectory of COVID units within designated facilities	4 (5%)	0 (0%)	4 (57%)	0 (0%)	“*I even started to wonder, what’s the point of our hot zone? To mobilize so much staff [] for about the equivalent of 8 patients.*” *(manager)*
Promote interdisciplinary collaboration	3 (3%)	1 (2%)	2 (29%)	0 (0%)	“*The good things, what I find that came out of the pandemic, is everything related to breaking down professional barriers, being able to be flexible in everyone’s roles, within the limits of the professional framework, of course.*” *(staff member)*
Equip rooms with better communication equipment	1 (1%)	1 (2%)	0 (0%)	0 (0%)	“*Patients who spent two months in their room, it happened. It’s terrible for any patient. But a stroke case that we isolate, everything related to social relationship, communication: we know the cognitive impacts it can have.*” *(manager)*”
Implement remote care—telehealth	1 (1%)	0 (0%)	1 (14%)	0 (0%)	“*Especially for external services [. . .], but even for internal services: organizing intervention plans with the family, communicate*” *(manager)*

*Note.* The table presents the 12 key recommendations derived from consultations with stakeholders in inpatient rehabilitation facilities during the COVID-19 pandemic. The recommendations are coded by their raw (N) and relative (%) frequency for each group: staff members, managers, and patients, with illustrative quotes.

Process analyses demonstrated substantial variations in care delivery. Length of stay differed markedly between groups, with COV+rehab nosocomial patients requiring extended stays (79 days) and COV+prior patients having shorter stays (30 days) compared to pre-pandemic averages (53 days). Both quantitative and qualitative data indicated insufficient care delivery, with patients consistently requesting increased therapy frequency. Discharge planning challenges were reported across unit types, characterized by patient unpreparedness and limited community support. Inter-professional challenges and expertise gaps were uniquely reported in COVID units, leading to recommendations for enhanced interdisciplinary collaboration. Two critical process-related themes emerged from stakeholder consultations: (1) the need to reassess COVID unit designation within facilities throughout different crisis phases, and (2) the importance of adapting infection prevention protocols to local facility contexts rather than applying acute or long-term care directives uniformly. Assessment practices were significantly impacted, with all COVID-affected groups showing decreased use of standardized performance tools compared to pre-COVID periods. Healthcare professionals across both unit types reported limited ability to implement evidence-based assessment and intervention in confined spaces.

**Outcome** measures showed alignment between quantitative and qualitative findings, with both COVID groups showing reduced FIM scores at discharge compared to pre-pandemic benchmarks. This echoes how the identified structural and process challenges may have impeded patient progress during the pandemic period. Quantitative data additionally showed elevated hospital readmission rates for COV+rehab patients (42% vs pre-pandemic 20%) and deliriums, which highlights the medical instability of this population. While not captured in medical records, stakeholder consultations revealed substantial unmet psychosocial needs across unit types, with higher prevalence in COVID units, leading to unanimous recommendations for addressing isolation and boredom.

### Priority Recommendations Identified by Stakeholders

From the original list of 7 patients invited to the consensus workshop, 4 could not be reached by phone. Three expressed interest in participating, but 1 had a medical appointment and another declined because her family member was on vacation at the time of the workshop and she did not wish to participate alone. In the end, 2 patients attended, each accompanied by a family member. Two rehabilitation professionals (an occupational therapist and a physiotherapist) and 4 managers were also present.

Through the iterative consensus process, these stakeholders consensually identified 5 priority recommendations that could inform decision-makers in the rehabilitation care system about crisis management. The remaining statements were considered lower priority and were discarded.

1. Create a hot zone environment favorable to therapeutic activities: Encourage physical activity and daily-life situations through technology and enriched environments.
2. Encourage adapted local management: Proximity management can consider the local resources and health situation to maintain client-program standards.
3. Value families: Reinforce families participation, especially during crucial transitions in the care trajectory; strike a balance between health prevention and psychosocial security.
4. Promote socialization: Fight isolation through technology and adapted spaces in the rehabilitation facility and provide psychosocial support.
5. Ensure a safe care trajectory: Develop specific rehabilitation guidelines and prepare for discharge by integrating families and community resources when available.

## Discussion

### Impact of Infection Timing on Rehabilitation Process and Outcomes

In accordance with our initial hypothesis and broader literature that documenting the general systemic impact of COVID-19 on body structure and function^6,9,28^: COVID-19 patient groups showed significantly lower functional autonomy compared to pre-pandemic levels. This study further contributes to existing knowledge by demonstrating that the timing of COVID-19 infection significantly influenced patients’ rehabilitation trajectories and length of stay, even after controlling for confounding factors such as characteristics at admission. During the critical pandemic period, patients who tested positive to COVID-19 (the COV+prior group of this study) were transferred to a designated IRF for an isolation period ranging from 10 to 14 days.^29^ During this short stay, they also received fewer dose of intervention (approximately 1 h per day vs the recommended 3 h daily, 5 days weekly).^3^ Conversely, patients who contracted COVID-19 during their stay (COV+rehab) experienced greater medical instability, including delirium and rehospitalization, resulting in extended rehabilitation periods and requiring more diverse disciplinary involvement (including psychologists and social workers). This pattern aligns with documented increases in healthcare resource utilization and hospital readmission rates among those acutely infected.^30,31^ Previous research also has shown that nosocomial SARS-CoV-2 infection during neurological rehabilitation reduces FIM scores (up to 8.9 points) after controlling for FIM at admission, age, sex, and morbidity index.^13^ Our study also adds new insight, as the COV+rehab group displayed lower admission FIM scores despite similar comorbidity profiles, suggesting that more vulnerable patients may have faced higher risk of contracting COVID-19 during their stay, further compounding their pre-existing conditions.^32,33^ Despite these rehabilitation challenges, our results align with recent meta-analyses,^9,34^ confirming that COVID patients can achieve meaningful recovery when given adequate support and time.

### Impact of the Pandemic Beyond the Disease

Our findings highlight the widespread effects of pandemic measures beyond direct COVID-19 infection. Qualitative data revealed that individuals who never contracted the virus were significantly impacted by the health crisis context. Through the Post-Acute Care (PAC) framework, which expands quality constituents of rehabilitation to include environmental context, we observed that preventive isolation measures and nosocomial outbreaks affected both COVID and non-COVID units alike. The cessation of family visits and social interaction emerged as the most detrimental impact, supporting findings from previous studies: isolation is well-documented to negatively affect patient morale, motivation, and coping behavior, increasing anxiety and depression.^15,16,35^ Stakeholders consistently reported that isolation impacted rehabilitation processes and outcomes—an aspect that cannot be fully assessed through our current methodology but has been explored in previous qualitative studies involving healthcare professionals and stroke patients.^36^ This underscores how the experience of a health crisis scenario itself can hinder functional gain, regardless of infection status^14^

Care coordination emerged as another significant challenge affecting all groups during the pandemic’s critical first year. Patients and families reported inadequate outpatient and community support following discharge from specialized inpatient rehabilitation units. The cessation of family visits negatively affected patient trajectories, as caregivers typically serve as crucial liaisons between medical teams and patients.^37^ This situation contradicts level A evidence supporting post-stroke interventions that combine skill-building with psycho-educational strategies^1^— approaches requiring direct and frequent contact. Broader macrosystemic studies of the stroke care continuum confirm that evidence-based standards^3^ were disrupted, with fewer referrals to IRFs during the pandemic^38^ and a 40% decline specifically among severe stroke patients.^39^

### Insights From Mixed Methods Design

Per design, our mixed-methods approach yielded complementary insights that would have remained uncaptured through either quantitative or qualitative approaches alone.^26^ While quantitative data revealed distinct trajectories for patients who acquired COVID-19 during rehabilitation—characterized by increased medical instability and longer stays—these patterns were not prominently featured in stakeholder accounts. This discrepancy likely stems from healthcare providers’ unit-specific experiences, potentially limiting their ability to observe infection timing-related patterns across the system. Conversely, traditional rehabilitation quality indicators documented in medical records—such as intervention frequency and length of stay—fail to capture the full spectrum of factors influencing recovery outcomes.^40^ This finding underscores the value of integrating multiple data sources when investigating complex healthcare delivery challenges during health crises.^41^

### Practical Guidelines for Maintaining Care Quality During Crises

This study presents the first comprehensive set of recommendations based on mixed-methods analysis incorporating multiple stakeholder perspectives during a contemporary healthcare crisis. While previous research has typically relied on single stakeholder groups, our consensus-based findings both validate and extend existing knowledge through 5 key recommendations for maintaining rehabilitation quality during healthcare crises. Existing literature provides guidance on implementing the 5 evidence-informed recommendations, offering decision-makers practical actions that can be integrated into crisis management contingency plans:

1- Psychosocial distress was identified as the predominant concern across all stakeholder groups, significantly impacting rehabilitation processes and outcomes. Healthcare facilities must carefully consider ethical implications when making decisions about family presence.^42^ Family involvement in day-to-day decision-making and care participation remains essential, even during health crises.^37^ To address these concerns, facilities can implement tailored infection prevention and control recommendations that balance safety with visitation and social activities.^16,43^ Several strategies effectively mitigate isolation’s impact, including technology training, increased staff social engagement, and environmental modifications in rehabilitation centers (eg, creating spaces for safe interaction among patients and family members).^43-45^
2- Rehabilitation intervention must be maintained despite isolation constraints. Evidence suggests patients should maintain or increase exercise frequency and intensity to counteract reduced physical activity during quarantines and room confinements.^37,46^ Telerehabilitation represents a valuable tool to support these efforts by providing at-home support to reinforce strategies learned in inpatient rehabilitation facilities.^47^
3- While healthcare crises may necessitate restructuring care delivery, such changes should not compromise established stroke care standards critical for optimal recovery timing.^3,48^ Our findings emphasize that maintaining safe care continuity requires comprehensive discharge planning that accounts for family resources and caregiver preparedness.^16,43^ Globally, facilities developed various approaches—some merged acute and rehabilitation care into unified units^49^ while others repurposed non-rehabilitation sites like hotels and community centers for COVID-19 short-term recovery.^50,51^ These diverse contingency approaches warrant systematic evaluation.
5- Healthcare systems require adaptive decision-making processes that respond to local contexts to meet specific patient needs while building crisis resilience. Previous studies have emphasized the need for flexible leadership to address evolving needs during different crisis stages^52-56^ During the COVID-19 pandemic, healthcare workers across European care services reported that infection prevention guidelines often failed to consider local contexts, creating conflicts with their professional values.^57^

### Limitations and Future Directions

Several limitations warrant consideration in interpreting our findings. From a quantitative perspective, the study’s generalizability is constrained by its finite sample size.^58^ Data representativity presented another challenge. We found particularly high rates of missing data for FIM post-discharge at 33% for COV+prior and 30% for COV+rehab compared with 19% for the PRECOVID reference group, which suggests that the data were not always missing at random. In consequence, the FIM post was not used in the multivariate model. This limited the full scope of our imputation approach.^59^ A previous investigation^60^ documented widespread issues with medical chart completeness and accuracy during the COVID-19 pandemic, partially attributable to infection control protocols that restricted documentation materials in COVID-positive patient rooms, thereby affecting both immediate data recording and standardized assessment procedures. In itself, this finding is significant as it demonstrates how the pandemic disrupted the guideline process component of the PAC-rehab quality framework. This disruption manifested through reduced use of standardized assessments recommended by stroke guidelines^2^ across all pandemic-admitted groups compared to the pre-COVID group. These findings align with concerns expressed by professionals and managers about compromised care standards due to equipment limitations, particularly in COVID units but also affecting non-COVID units.

Our qualitative analysis also faced certain constraints, especially regarding stakeholder recruitment and representativeness. The patient sample consisted exclusively of home-dwelling individuals, potentially overlooking the experiences of those with less favorable outcomes who may have been transferred to long-term care facilities or remained hospitalized. Additionally, our recruitment criteria required participants to communicate in English or French or have family support available, which may have excluded patients with severe communication impairments or without adequate support systems. Furthermore, this study did not capture perspectives from professionals working in alternative COVID post-acute settings such as community centers and hotels,^50,51^ limiting the diversity of rehabilitation experiences represented in our findings. The convenience sampling approach, while practical during pandemic restrictions, may have introduced selection bias by overrepresenting certain demographic groups or those with more positive experiences. However, the achievement of theme saturation during coding suggests adequate sample size for addressing our research questions. While lived experiences cannot establish direct causality for chart indicators, the integration of subjective perspectives with objective data provides valuable insights for future pandemic preparedness and crisis management directives. The TRIAGE method further allowed for a systematic integration of diverse stakeholder perspectives while facilitating meaningful synthesis of individual and collective expertise. In addition, the absence of validated questionnaires or interview guides specifically designed for assessing rehabilitation quality during healthcare crises highlights the need for future development in this area.

From a broader perspective, the fact that data were collected from a single specific geographic area represents another limitation. Given that rehabilitation care pathways and pandemic responses varied across healthcare systems globally, despite sharing common delivery aspects, similar retrospective analyses in other countries would enhance our understanding of effective crisis management strategies. For example, while our study is based in Quebec’s universal healthcare system, implementation in insurance-based systems will need tailored approaches. Although specific implementation strategies may vary across settings, the core neurorehabilitation principles tackled by our consensus-based recommendations—including interdisciplinary teamwork, safe care trajectory, and functional goal-oriented therapy—remain fundamental to quality rehabilitation delivery regardless of healthcare system structure. Implementation of our 5 consensus recommendations may face several barriers in future health crises, such as space and equipment limitations for isolated therapeutic zones; rigid command structures for local management; conflicts between infection control and family presence; resource constraints and technology access inequities. Future research should address these potential barriers by developing implementation frameworks and system-specific toolkits for crisis preparedness that can be adapted to different healthcare contexts and resource levels.

## Conclusion

Overall, this study revealed significant impacts of the COVID-19 pandemic on inpatient neurorehabilitation care delivery, affecting functional outcomes, but also personnel, equipment, and organizational dimensions of quality. The mixed-methods design yielded complementary insights. Quantitative analysis demonstrated distinct trajectories between patient groups. While non-COVID units maintained pre-pandemic performance indicators, COVID-positive patients exhibited markedly different care patterns based on infection timing. Rehabilitation-acquired COVID-19 cases (COV+rehab) demonstrated less efficient trajectories, characterized by extended lengths of stay, increased complications (delirium and relapse), and more diverse interdisciplinary involvement. Conversely, patients COVID-positive at admission (COV+prior) experienced abbreviated stays with reduced treatment intensity and interdisciplinary involvement. This finding warrants for careful interpretation of rehab trajectory depending on the timing of infection. Qualitative investigation revealed common challenges across all units, particularly regarding psychosocial support and discharge planning complexities. COVID units faced additional challenges, with patients reporting care insufficiencies while staff identified inadequate rehabilitation environments and resource constraints.

This study proved the value of integrating multiple data sources in understanding complex healthcare delivery challenges during crises, to reflect the complex interplay between infection timing, medical instability, and stringent institutional protocols in managing a healthcare crisis patients in rehabilitation settings. Through data integration, 5 consensus-based recommendations were developed, emphasizing the need for rehabilitation-specific crisis protocols that prioritize rehabilitation settings, integrate family involvement and maintain an environment conducive to social, cognitive, and physical rehabilitation.
